# Trends and Determinants of Non-Utilization of Modern Contraception in Ekiti State, Nigeria: A Ten-Year Review

**DOI:** 10.34763/jmotherandchild.20232701.d-22-00067

**Published:** 2023-08-07

**Authors:** Oluwafunmilayo Oluwadamilola Ibikunle, Tope Michael Ipinnimo, Ayobami Oyekunle Afape, Austine Idowu Ibikunle, Caroline Ajoke Bakare, Babatunde Ajidagba, Demilade Olusola Ibirongbe, Esther Opeyemi Ajidahun, Kabir Adekunle Durowade, Adebowale Femi Akinwumi, Ayokunle Faniku, Babatunde Adelekan

**Affiliations:** Ekiti State Ministry of Health & Human Services, Ado-Ekiti, Nigeria; Department of Community Medicine, Federal Teaching Hospital, Ido-Ekiti, Nigeria; State Primary Health Care Development Agency, Kaduna, Nigeria; State Primary Health Care Development Agency, Ado-Ekiti, Nigeria; Independent Researcher, Luton, Bedfordshire, UK; Department of Community Medicine, University of Medical Sciences, Ondo, Nigeria; University of Ilorin Teaching Hospital, Ilorin, Nigeria; Department of Community Medicine, Ekiti State University Teaching Hospital, Ado-Ekiti, Nigeria; Centre for Health Economics and Development, Abuja, Nigeria; United Nations Population Fund, Abuja, Nigeria

**Keywords:** Contraceptive prevalence rate, modern contraceptives, unmet needs, family planning, Ekiti State

## Abstract

**Background:**

An increase in correct usage of modern contraception is vital in reducing the maternal mortality ratio and Under-5 mortality, leading towards the achievement of Sustainable Development Goal 3. Our study examined the trends and factors affecting non-utilization of modern contraceptives over a 10-year period in Ekiti State, Nigeria.

**Methodology:**

This study used data from three consecutive National Demographic Health Surveys (NDHS) – 2008, 2013, and 2018 – with a weighted sample size of 1,357 women of reproductive age (15-49 years). Data on contraceptive use on these women, provided by the NDHS, were extracted and analysed using IBM SPSS version 25. The sample was weighted to adjust for disproportionate sampling and non-response. Pearson's chi-square and binary logistic regression were used to assess the factors associated with non-utilization of modern contraceptives.

**Results and findings:**

The mean age of the women was 30 years. Modern contraceptive use increased from 13.1% in 2008 to 23.0% in 2018, while unmet need for modern contraceptives decreased from 84.8% in 2008 to 75.4% in 2018. Identified predictors of non-utilization of modern contraceptive were age 20–24 years [aOR=0.33, 95%CI=0.19–0.59], 25–29 years [aOR=0.34, 95%CI=0.18–0.64], 30–34 years [aOR=0.46, 95%CI=0.22–0.94], 35–39 years [aOR=0.29, 95%CI=0.14–0.61] and 40–44 years [aOR=0.37, 95%CI=0.17–0.80] compared to age 15–19 years; living in urban areas [aOR=0.72, 95%CI=0.53–0.98] compared to in rural areas; higher level of education [aOR=0.46, 95%CI=0.21–0.98] compared to no education; and desire for more children [aOR=0.48, 95%CI=0.32–0.73] compared to not wanting more children.

**Conclusion:**

Although contraceptive usage increased over time, the factors associated with non-utilization were being an adolescent, living in a rural area, lower level of education, and desire for more children.

## Introduction

In achieving the sustainable development agenda, the contraceptive prevalence rate (CPR) has been identified as a vital indicator due to its immense benefits [1]. Family planning helps couples and individuals realize their basic right to decide freely and responsibly if, when, and how many children to have, resulting in improvements in sexual and reproductive health outcomes for the woman, the survival and growth of the child, and the general wellbeing of the family [2,3,4].

Contraceptive use helps women in the control of the number, interval, and timing of pregnancies and births, thereby reducing unwanted pregnancy, demand for unsafe abortion, and maternal mortality and morbidity [1,5]. In addition, the growing use of contraceptive methods has resulted in improvements in schooling and economic outcomes, especially for girls and women, through securing the wellbeing and autonomy of women; this has enhanced the health and development of communities and nations [6,7].

It is an established fact that increased contraceptive use and reduced unmet need for contraception are central to improving maternal health and reducing child mortality [8,9]. However, modern contraceptive use among women of reproductive age only increased slightly globally over 15 years, from 54% in 1990 to 57% in 2015 [10,11] The African region also witnessed a minimal rise from 23.6% to 28.5% [11], with no noticeable increase in West Africa (especially Nigeria) and Middle Africa, areas where modern contraception use continues to be very low [8].

This very low contraceptive prevalence in most developing countries remains associated with high unmet need for family planning among sexually active women in these countries [8,9,10]. 214 million women of reproductive age in developing countries who want to avoid pregnancy are not using a modern contraceptive method [11]. Furthermore, the unmet need for modern contraception in the 69 poorest countries (Nigeria included) – which account for 73% of all unmet need in developing countries – also increased from 153 million to 162 million women in 2008 – 2012 [8].

In Nigeria, the National Demographic Health Survey (NDHS) in 2018 placed the CPR at about 17%, which was slightly higher than the estimates from previous national surveys [12,13,14]. This data is supported by a community-based study conducted across Nigeria, which revealed that contraceptive prevalence among sexually active respondents was 14.8% for all methods, and 10.1% for modern methods [15]. Of the 45 million women of reproductive age in Nigeria, 15.7 million want to avoid pregnancy [9]. According to a national survey, the unmet need for family planning among women of reproductive age in the Southwest region is 17.8% [14]. In Ekiti State, one in every four women is currently using any of the modern contraceptive methods. Additionally, the unmet need for family planning remained the same over the years [12,13,14]. This lack of change exists despite huge investments for improved sexual and reproductive health of women through the use of contraceptives in recent times through strategic programs, including the Family Planning 2020 initiative, Saving One Million Lives Program for Results [16,17]. Several million women who desire contraceptives are not using it, especially in the 69 poorest countries (Nigeria included). In the developing world in general, and Nigeria in particular, where change is slow and the use of modern contraceptive methods is very low – and where unwanted pregnancy, unsafe induced abortion, high fertility rates, and STIs & HIV/AIDS are all very serious reproductive health problems – urgent and particularly careful examination to determine how to meet existing contraceptive needs is required [88].

There is a need to identify the trends in the non-utilization of modern contraceptive methods in Nigerian communities, and the factors associated with these trends. The description of trends of non-utilization of modern contraceptive methods in Ekiti State (on the sub-national level) is necessary for monitoring progress, or otherwise an understanding of associated factors at play might be different from those identified at the assessment of non-utilization at the national level over this period. The aim of this study is to examine the determinants of non-utilization of family planning and unmet needs of family planning in Ekiti State over a 10-year period so as to inform policy direction in improving contraceptive uptake among child-bearing-aged women in the State. The research questions are: 1) What is the trend of non-utilization of modern contraception over a 10-year period in Ekiti State; 2) What are the factors affecting non-utilization of modern contraception over a 10-year period in Ekiti State.

## Methodology

### Data Sources

This study analysed data from the National Demographic Health Surveys (NDHS) for 2008, 2013, and 2018, which resulted in a weighted sample size of 1,357 women of reproductive age. These were cross-sectional analyses of nationally representative secondary data. These surveys provide relevant health and social information (including maternal and child health, and trends in key health indicators in the population) to guide policy decisions. These surveys used the sampling frame to determine the enumeration areas (EAs), local government areas (LGAs), states, and zones in Nigeria as prepared in the 2006 Population Census of the Federal Republic of Nigeria. The sample used for this review was selected using a stratified three-stage cluster design, spread over rural and urban areas in Nigeria [14].

This study focuses on Ekiti State subset of the survey sample. Ekiti State is located in Southwest Nigeria with a projected population of 3.4 million [18]. Ekiti State comprises 16 Local Government Areas (LGAs) in three senatorial districts, namely Ekiti Central, Ekiti North, and Ekiti South. The inhabitants of the city are mainly Yoruba-speaking people and mostly Christians. There are 326 Primary Health Care (PHC) facilities, 22 general hospitals (including 3 Specialist Hospitals in each Senatorial District) and 3 tertiary health facilities. In addition, family planning services are offered across selected government health facilities. The total number of family planning facilities in Ekiti State is 154. PHC facilities have 131, secondary facilities have 21, and there are 2 in tertiary facilities.

### Outcome Variable

We extracted contraceptive use information provided by the NDHS about women of childbearing age (15 to 49), including those who were either currently married or in a sexual union. The primary outcomes investigated in this study are the non-utilization of modern contraceptives and the unmet need for contraceptives. The variable for non-utilization of modern contraceptives was derived from the question “current contraceptive use by methods,” for which the possible responses were “no method,” “folkloric method,” “traditional method,” and “modern method.” Contraceptive use was recoded into “non-modern contraception=1” for those who do not use modern contraceptives (i.e., no method, folkloric method, and traditional method), and “modern=0” for those using modern contraceptives. The variable for unmet need for contraceptive use was measured by computing the percentage of women (only those currently married or in a sexual union) who do not want to become pregnant, either for ending their childbearing or delaying their next pregnancy, and are not using a contraceptive method [19].

### Independent Variables

Important individual social determinants were considered in the analyses. A widely used model to explain individual determinants of healthcare utilization is Anderson's behavioral framework [20,21] which noted that an individual's use of a service (in this case, family planning) is considered to be a function of 3 components – predisposing factors (in this case, sociocultural characteristics of women of the reproductive age group which existed prior to, affecting the propensity to use, care); enabling determinants (here referring to the conditions which make family planning services available to women of reproductive age); and need factors (here referring to women of the reproductive age group related to the elements explaining the degree of care needed).

### Statistical analysis

The authors conducted descriptive, bivariate, and multivariate analyses. First, we used sample weighting to adjust for disproportionate sampling and non-response. For the descriptive analysis, Pearson's chi-square test was used to identify the association between the outcome variable and the independent variables in their categories. Study variables with a p-value <0.05 at the bivariate analysis level were considered for inclusion into the model. Lastly, we used a binary logistic regression model to assess the factors associated with non-utilization of modern contraceptive usage among women of childbearing age in Ekiti State at a 95% confidence interval (CI) with computed adjusted odds ratios (aORs). All the study data were analyzed using IBM SPSS version 25.

### Ethical approval

The datasets used in this research were population-based datasets freely available in the public domain. For confidentiality reasons, specific characteristics that could be used to identify participants in the study were excluded. MEASURE DHS/ICF International permitted the authors to use the datasets as a secondary study. Also, before the survey, the DHS project received ethical approval from Nigeria's National Health Research Ethics Committee (NHREC).

## Results

**Figure 1.**
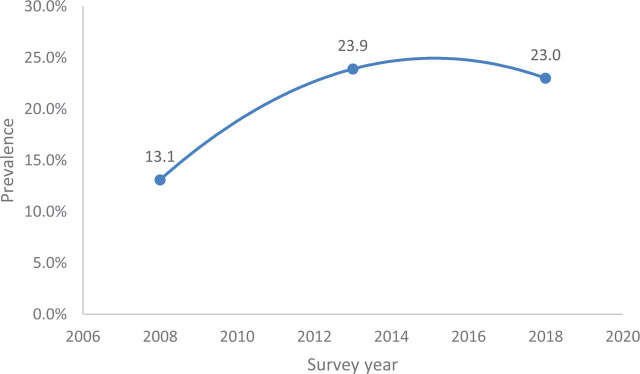
Trend of modern contraceptive use among women of childbearing age in Ekiti State: 2008–2018.

**Figure 2.**
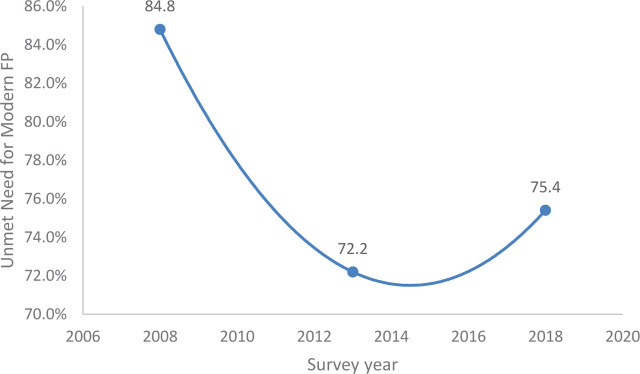
Trend of unmet need for modern contraceptive among married or sexually active women of childbearing age in Ekiti State: 2008–2018.

Concerning the trends of contraceptive non-utilization, we found that the modern contraceptive use among women of childbearing age 15 to 49 in Ekiti State, Nigeria increased from 13.1% in 2008 to 23.0% in 2018 (Fig. 1). Similarly, it was found out that the unmet need for modern contraceptive among married or sexually active women of childbearing age in Ekiti State Nigeria decreased from 84.8% in 2008 to 75.4% in 2018 (Fig. 2).

Selected determinants were assessed during the analysis; based on the pooled data, the mean age (±SD) of the women in the study was 29.6 (±10.1) years. Almost two-thirds were married, 57.9% had at least secondary education, and more than half of the women resided in urban areas. More than 82% practiced Christianity and only 17% were within the wealth quintile indicating poverty. About 82% claimed their distance to the nearest health facility was not a big problem, over 59% had been told about FP in a health facility, only 30.7% did not have a desire for more children, and only 2% were covered by insurance. More than half of the women claimed to have joint decisions with their partner on contraceptive usage, and only 19.2% of the women used modern contraceptives (Table 1).

**Table 1.** Socio-demographic characteristics of weighted sample population (NDHS 2008–2018)

	**2008**	**2013**	**2018**	**Pooled 2008–2018**

	**n (%)**	**n (%)**	**n (%)**	**n (%)**
**Women age**				
15–19	124 (22.3%)	71 (21.7%)	82 (17.5%)	278 (20.5%)
20–24	85 (15.3%)	49 (15.0%)	67 (14.1%)	201 (14.8%)
25–29	106 (19.1%)	56 (17.1%)	73 (15.4%)	235 (17.3%)
30–34	82 (14.7%)	44 (13.5%)	63 (13.3%)	189 (13.9%)
35–39	64 (11.5%)	36 (11.0%)	60 (12.6%)	160 (11.8%)
40–44	43 (7.7%)	37 (11.3%)	60 (12.6%)	140 (10.3%)
45–49	52 (9.4%)	34 (10.4%)	69 (14.5%)	155 (11.4%)
Mean (±SD)	28.5 ± 9.6	29.3 ± 10.2	31.1 ± 10.3	29.6 ± 10.1
**Place of residence**				
Urban	220 (39.6%)	262 (80.1%)	386 (81.3%)	868 (63.9%)
Rural	336 (60.4%)	65 (19.9%)	89 (18.7%)	490 (36.1%)
**Level of education**				
No education	42 (7.6%)	7 (2.1%)	24 (5.1%)	73 (5.4%)
Primary	96 (17.3%)	42 (12.8%)	70 (14.7%)	208 (15.3%)
Secondary	307 (55.2%)	195 (59.6%)	284 (59.8%)	786 (57.9%)
Higher	111 (20.0%)	83 (25.4%)	97 (20.4%)	291 (21.4%)
**Marital status**				
Never married	209 (37.6%)	119 (36.5%)	123 (25.9%)	451 (33.3%)
Married/Living with partner	333 (59.9%)	194 (59.5%)	326 (68.8%)	853 (62.9%)
Formerly married	14 (2.5%)	13 (4.0%)	25 (5.3%)	52 (3.8%)
**Religion**				
Christianity	459 (82.6%)	300 (91.7%)	365 (76.8%)	1124 (82.8%)
Islam	97 (17.4%)	27 (8.3%)	110 (23.2%)	234 (17.2%)
**Occupational status**				
Working	309 (56.0%)	204 (62.4%)	362 (76.45)	875 (64.7%)
Not working	243 (44.0%)	123 (37.6%)	112 (23.6%)	478 (35.3%)
**Wealth index**				
Poor	119 (21.4%)	4 (1.2%)	111 (23.3%)	234 (17.2%)
Middle	130 (23.4%)	55 (16.8%)	111 (23.3%)	296 (21.8%)
Rich	307 (55.2%)	268 (82.0%)	254 (53.4%)	829 (61.0%)
**Covered by health insurance**				
Yes	10 (1.8%)	9 (2.8%)	8 (1.7%)	27 (2.0%)
No	544 (98.2%)	317 (97.2%)	466 (98.3%)	1327 (98.0%)
**Distance to health facility**				
Big problem	112 (20.2%)	35 (10.7%)	95 (20.0%)	242 (17.9%)
Not a big problem	442 (79.8%)	291 (89.3%)	380 (80.0%)	1113 (82.1%)
**FP services available at the facility**				
Yes	91 (59.1%)	58 (64.4%)	134 (58.3%)	283 (59.7%)
No	63 (40.9%)	32 (35.6%)	96 (41.7%)	1919 (40.3%)
**Desire for more children**				
More children	382 (68.8%)	213 (65.1%)	273 (57.5%)	868 (64.0%)
Undecided	34 (6.1%)	16 (4.9%)	22 (4.6%)	72 (5.3%)
No more children	139 (25.0%)	98 (30.0%)	180 (37.9%)	417 (30.7%)
**Woman's autonomy in decision making for family planning**				
Respondent alone	97 (29.2%)	55 (28.4%)	60 (18.4%)	212 (24.9%)
Respondent and husband/partner	156 (47.0%)	85 (43.8%)	218 (66.9%)	459 (53.9%)
Husband/partner alone	79 (23.8%)	54 (27.8%)	48 (14.7%)	181 (21.2%)
**Contraceptives Use**				
Modern method	73 (13.1%)	78 (23.9%)	109 (23.0%)	260 (19.2%)
Non modern method	483 (86.9%)	248 (76.1%)	365 (77.0%)	1096 (80.8%)

The bivariate analysis of the selected determinants indicated that age, place of residence, level of education, marital status, wealth index, and desire for more children were significant factors associated with non-utilization of modern contraceptives among women of reproductive age in Ekiti State, Nigeria (Table 2). The results show that non-modern contraceptives use was significantly higher among adolescents aged 15–19 (91.4%; p-value – 0.001), rural dwellers (85.1%; p-value – 0.003), women with no education (86.3%; p-value – 0.017), formerly married women (86.5%; p-value – 0.009), poor women (85.5%; p-value – 0.010) and women who desired more children (83.9%; p-value – 0.001). Table 2 further shows the binary regression results on the factors associated with non-utilization of modern contraceptive use among women of childbearing age in Ekiti State. In the adjusted model, age was associated with non-utilization of modern contraceptives; women aged 20–24, 25–29, 30–34, 35–39, and 40–44 years respectively had significantly lower odds [aOR=0.33, 95% CI= 0.19–0.59] [aOR=0.34, 95% CI= 0.18–0.64] [aOR=0.46, 95% CI= 0.22–0.94] [aOR=0.29, 95% CI= 0.14–0.61] [aOR=0.37, 95% CI= 0.17–0.80] of non-utilization of modern contraceptives compared to adolescents aged 15–19 years. In addition, those who lived in urban areas had significantly lower odds [aOR 0.72, 95% CI= 0.53–0.98] of non-utilization of modern contraceptives than those living in rural areas. Furthermore, women who attained a higher level of education had significant lower odds [aOR 0.46, 95% CI= 0.21–0.98] of non-utilization of modern contraceptives than those with no education. Also, women who still desired more children had significantly lower odds [aOR=0.48, 95% CI= 0.32–0.73] of non-utilization of modern contraceptives compared to women who did not want more children.

**Table 2.** Bivariate and multivariate analyses of non-modern contraceptive use among women of reproductive age (15–49) in Ekiti State (NDHS 2008–2018) (Weighted sample)

**Variables**	**Types of Contraceptives**		**Bivariate Analysis (chi-square test)**	**Binary Logistic Regression**

	**Non-modern (%)**	**Modern (%)**	**P value**	**AOR (95%CI)**
**Women age**			0.001^*^	
15–19	254 (91.4%)	24 (8.6%)		1
20–24	157 (78.5%)	43 (21.5%)		0.33 (0.19–0.59)^*^
25–29	183 (77.9%)	52 (22.1%)		0.34 (0.18–0.64)^*^
30–34	155 (82.0%)	34 (18.0%)		0.46 (0.22–0.94)^*^
35–39	116 (72.5%)	44 (27.5%)		0.29 (0.14–0.61)^*^
40–44	104 (73.8%)	37 (26.2%)		0.37 (0.17–0.80)^*^
45–49	128 (83.1%)	26 (16.9%)		0.62 (0.27–1.44)
**Place of residence**			0.003^*^	
Urban	681 (78.5%)	187 (21.5%)		0.72 (0.53–0.98)^*^
Rural	416 (85.1%)	73 (14.9%)		1
**Level of education**			0.017^*^	
No education	63 (86.3%)	10 (13.7%)		1
Primary	173 (83.6%)	34 (16.4%)		0.77 (0.35–1.69)
Secondary	643 (81.8%)	143 (18.2%)		0.55 (0.26–1.15)
Higher	217 (74.6%)	74 (25.4%)		0.46 (0.21–0.98)^*^
**Marital status**			0.009^*^	
Never married	383 (84.9%)	68 (15.1%)		1
Married/Living with partner	668 (78.3%)	185 (21.7%)		1.23 (0.79–1.92)
Formerly married	45 (86.5%)	7 (13.5%)		1.95 (0.77–4.90)
**Religion**			0.299	
Christianity	902 (80.3%)	221 (19.7%)		-
Islam	194 (83.3%)	39 (16.7%)		-
**Occupational status**			0.789	
Working	705 (64.5%)	170 (65.4%)		-
Not working	388 (35.5%)	90 (34.65)		-
**Wealth index**			0.010^*^	
Poor	200 (85.5%)	34 (14.5%)		1
Middle	249 (84.4%)	46 (15.6%)		0.91 (0.56–1.50)
Rich	648 (78.3%)	180 (21.7%)		0.75 (0.48–1.17)
**Covered by health insurance**			0.060	
Yes	1076 (81.1%)	251 (18.9%)		-
No	18 (66.7%)	9 (33.3%)		-
**Distance to health facility**			0.965	
Big problem	195 (80.9%)	46 (19.1%)		-
Not a big problem	900 (80.8%)	214 (19.2%)		-
**FP services available at the facility**			0.229	
Yes	232 (82.0%)	51 (18.0%)		-
No	148 (77.5%)	43 (22.5%)		-
**Desire for more children**			0.001^*^	
More children	728 (83.9%)	140 (16.1%)		1
Undecided	60 (82.2%)	13 (17.8%)		0.87 (0.45–1.69)
No more children	309 (74.3%)	107 (25.7%)		0.48 (0.32–0.73)^*^
**Woman's autonomy in decision making for family planning**			0.803	
Respondent alone	165 (77.8%)	47 (22.2%)		-
Respondent and husband/partner	358 (77.8%)	102 (22.2%)		-
Husband/partner alone	145 (80.1%)	36 (19.9%)		-

*Sources*: NDHS 2008, 2013, 2018,

* *p* < 0.05 were statistically significant,

AOR= Adjusted Odd Ratio, CI= Confidence Interval, 1= Reference Category. – Excluded variables

## Discussion

With respect to the trend of non-utilization of modern contraception in Ekiti State, this study shows that modern contraceptive use steadily increased from 13.1% in 2008 to 23.0% in 2018. This is similar to the findings of a study among reproductive-aged women in Kenya where contraceptive use increased from 24.0% to 42.6% over a period of two decades [22]. Nepal recorded an increase of more than threefold over 20 years in contraceptive use among female adolescents [23]. In addition, Ethiopia recorded an increase in contraceptive use among young women between 2000 and 2011 [3]. However, this is in contrast to a study done in Ghana among adolescents, which found contraceptive use declined from 22.1% in 2003 to 20.4% in 2014 [25]. The reason for the rise in contraceptive use over the decade may be due to the increased enlightenment and awareness campaign program on contraceptives and family planning during this period. There was also integrated supportive supervision of healthcare workers administering contraceptive commodities in Ekiti State, sponsored by Saving One Million Lives Program for Results, during this period.

Furthermore, the unmet need for modern contraceptives decreased from 84.8% in 2008 to 75.4% in 2018, which is similar to the decline in the unmet needs among women in developing countries between 2008 and 2012 [26]. Proper education against barriers of carefree attitudes, religious prohibition, myths, misconceptions, fears, and hearsay can help to reduce unmet need [1]. It is known that increased contraceptive use and reduced unmet need for contraception are central to improving maternal health and reducing child mortality [25,26].

Concerning the factors affecting non-utilization of modern contraception in Ekiti State, this study found a lower utilization of modern contraceptives among younger women. This is consistent with a study in Ghana, where adolescents used more non-modern contraceptives [25]. One possible reason could be that adolescents who were not married may face several barriers in accessing and using modern contraceptives, because sexual activity is only considered acceptable within marriage in many African settings, including Ekiti State, Nigeria. A study in Myanmar similarly found out that, interestingly, never-married female youth did not use contraception, and they were reported as sexually inactive [27]. The possibility is that, just like the adolescents in Ghana, some unmarried youth might be reluctant to consult with healthcare providers regarding contraceptive services due to stigmatization. Culture and tradition seem to play a very significant role in preventing the utilization of modern contraceptives among never-married youth.

In addition, there was a lower utilization of modern contraceptives among women in rural areas, which is not surprising, as those who lived in rural areas are less likely to have formal education, and more likely to be poor, therefore having lower modern contraceptive use. Women with no formal education were more likely to earn less and live in rural areas. Educated women were more aware and more likely to understand the importance of contraceptive use. This finding is similar to that of a Nigerian study that showed a higher contraceptive use in urban areas [15]. It is no surprise that rural dwellers had a higher prevalence of unwanted pregnancies in the study. It was also discovered that contraceptive prevalence is directly associated with educational status. This finding may be due to the fact that educated women will tend to comprehend the messages about contraception during awareness campaigns. It may be necessary to develop special communication strategies to reach out to women in rural areas during such enlightenment campaigns [15]. Additionally, women in urban areas may be more exposed and have better access to media, and they may have visited nearby health facilities to obtain information on modern contraception and other reproductive health services. The desire for more children is another significant factor in this study. There was an increase in the non-utilization of modern contraceptives among women who desire to have more children. This is similar to a study done among in-union women in Nigeria, where it was discovered that the desire for more children was a significant factor in reducing modern contraceptive use [1]. In addition, an average sexually active in-union woman desired seven children, but this varied based on individual characteristics [1]. Furthermore, in a study done among women in Ise-Ekiti, Ekiti State, it was reported that the reason for non-utilization of modern contraceptives was the women desiring more children [28]; this can be explained by the culture and mindset about child-bearing of people living in sub-Saharan Africa. There is a strong desire for a large family size, desire for a specific number of children of a particular gender, and a sense of accomplishment that is derived from having many children [29].

This study is limited by the use of secondary data from NDHS, as errors transmitted initially into the data will affect the outcome of this study. The strength of this study lies in the large sample size used for the study, as this is large enough to show significant association for advanced statistical analysis.

## Conclusion

In conclusion, our study showed that there was a steady increase in modern contraceptive use and a decline in the unmet need for modern contraceptives in Ekiti State between 2008 and 2018. Modern contraceptive utilization was found to be reduced among younger women (especially adolescents), those living in rural areas, those with lower levels of education, those of low socioeconomic class, and those who desire more children. Young people should be targeted in order to inculcate a positive attitude towards family planning, including the use of Emergency Contraceptive Pills to prevent unwanted pregnancy. Government policies and targeted behavioural change communications strategies that will increase contraceptive use among high-risk groups must be encouraged.

### Key Points

- The use of modern contraceptives increased during the 10-year period.
- The unmet need for modern contraceptives decreased during the 10-year period.
- The identified predictors in the study were age, place of residence, level of education, wealth index, and desire for more children.
- The public health implication is the improvement in maternal health and, invariably, child health in Ekiti State over the 10-year period.
